# Performance Analysis of Integrated Wireless Sensor and Multibeam Satellite Networks Under Terrestrial Interference

**DOI:** 10.3390/s16101711

**Published:** 2016-10-14

**Authors:** Hongjun Li, Hao Yin, Xiangwu Gong, Feihong Dong, Baoquan Ren, Yuanzhi He, Jingchao Wang

**Affiliations:** 1 College of Communications Engineering, PLA University of Science and Technology, Nanjing 210007, China; 2 Institute of China Electronic System Engineering Corporation, 13 Dacheng Rd., Beijing 100141, China; yinhao1@263.net (H.Y.); yiheng689@126.com (X.G.); dfh_sinlab@163.com (F.D.); renbq88@sohu.com (B.R.); heyz_8280@163.com (Y.H.); wangwangjingchao@126.com (J.W.); 3 Equipment Academy, Beijing 101416, China

**Keywords:** integrated wireless sensor and multibeam satellite networks, terrestrial interference, optimal capacity, minimum mean square error, Haar approximation, Rician fading

## Abstract

This paper investigates the performance of integrated wireless sensor and multibeam satellite networks (IWSMSNs) under terrestrial interference. The IWSMSNs constitute sensor nodes (SNs), satellite sinks (SSs), multibeam satellite and remote monitoring hosts (RMHs). The multibeam satellite covers multiple beams and multiple SSs in each beam. The SSs can be directly used as SNs to transmit sensing data to RMHs via the satellite, and they can also be used to collect the sensing data from other SNs to transmit to the RMHs. We propose the hybrid one-dimensional (1D) and 2D beam models including the equivalent intra-beam interference factor *β* from terrestrial communication networks (TCNs) and the equivalent inter-beam interference factor *α* from adjacent beams. The terrestrial interference is possibly due to the signals from the TCNs or the signals of sinks being transmitted to other satellite networks. The closed-form approximations of capacity per beam are derived for the return link of IWSMSNs under terrestrial interference by using the Haar approximations where the IWSMSNs experience the Rician fading channel. The optimal joint decoding capacity can be considered as the upper bound where all of the SSs’ signals can be jointly decoded by a super-receiver on board the multibeam satellite or a gateway station that knows all of the code books. While the linear minimum mean square error (MMSE) capacity is where all of the signals of SSs are decoded singularly by a multibeam satellite or a gateway station. The simulations show that the optimal capacities are obviously higher than the MMSE capacities under the same conditions, while the capacities are lowered by Rician fading and converge as the Rician factor increases. *α* and *β* jointly affect the performance of hybrid 1D and 2D beam models, and the number of SSs also contributes different effects on the optimal capacity and MMSE capacity of the IWSMSNs.

## 1. Introduction

Wireless sensor networks (WSNs) have been highly developed, which have lowered costs along with the rapid development of wireless communications and electronics technologies, and are widely used in various areas [1]. The researchers focused on different issues appropriate for particular WSN applications. WSNs have been applied to many fields and even cannot be replaced, in the areas of target tracking [2], disaster warning [3], wildlife monitoring [4], military applications [5,6], health monitoring [7], monitoring and navigation [8,9,10], etc. Obviously, the communications are very important to WSNs, as the sensing information must be transmitted to the remote monitoring hosts (RMHs).

Multiple input and multiple output (MIMO) has been regard as the important technology application for the future high data rate wireless communication networks [11,12]. Therefore, [13,14,15,16,17,18] investigated the MIMO transmission for the WSNs. The work in [13] studied the cooperative MIMO (C-MIMO) technology and adopted the low density parity check (LDPC) code as the error-correcting code, while [14,15] analyzed the virtual MIMO (V-MIMO) for WSNs. The former configured the minimum energy consuming route and satisfied the end-to-end data rate and bit error rate (BER) requirements, and the latter analyzed the impact of the modulation techniques for the cooperative V-MIMO of WSNs. The work in [16] proposed an adaptive multi-node MIMO transmission to improve the transmission reliability and capacity of mobile sink nodes for the WSNs. The work in [17] presented an efficient and adaptive mutual authentication framework for the heterogeneous WSNs. Meanwhile, [18] introduced the nonlinear MIMO networks with the characteristics of low cost and low complexity for the WSNs.

However, the research in [13,14,15,16,17,18] has mainly focused on the terrestrial network applications, such as the lack of terrestrial networks coverage or where terrestrial networks were challenged by the operating environment, e.g., forest, wilderness and military environments. Since the multibeam satellite communication networks have the advantages of global coverage, unrestricted geography, low cost, quick response [19], and so on, satellite-based sensor networks have been investigated for various application scenarios [20,21,22]. The satellite networks can be considered as an important auxiliary to WSNs and may be used as the transmission link of the integrated wireless sensor and multibeam satellite networks (IWSMSNs). Multibeam satellite networks share some of the same characteristics, such as low cost, low complexity and unrestricted geography, which are very suitable for WSNs.

Like terrestrial WSNs, the satellite-based WSNs can also be applied to many fields, such as target detection and tracking, vehicular navigation and environmental monitoring [23,24,25]. The work in [23] proposed a hybrid architecture for which the first category performed detection and tracking functions and the second category transmitted sensing data through the satellite communication networks. The work in [24] proposed a data fusion technique based on radial basis function neural networks, which integrated a global positioning network with terrestrial sensor networks. The work in [25] presented a sink selection algorithm for the satellite-based environmental monitoring networks against the fading, noise and component failure.

In particular, [26] investigated the architectures and scenarios for satellite-based WSNs and the potential role of satellite/space networks might play in certain WSN applications to provide integrated configurations. Then, [27] addressed four integrated sensor-satellite network architectures and analyzed the performance of packet loss rate, average end-to-end packet delay and overall energy consumption. The work in [28] evaluated the remote sensor and satellite network capacity and attempted to optimize the integrated wireless sensor and satellite network schedules. Furthermore, [29,30] investigated the applications of near-space and high altitude platform (HAP)/satellite for the WSNs. The work in [29,30] proposed a novel integrated HAP/satellite and designed a link-state advertisement to estimate the link state information; ultimately, an innovative adaptive algorithm was addressed to obtain the energy-efficient transmission; whilst introducing several near-space vehicle-based radar configurations, which seems to offer a lower cost, such as near-space passive bistatic radar and high-resolution wide-swath synthetic aperture radar.

However, the transmission research of integrated wireless sensor and satellite networks [23,24,25,26,27,28,29] did not consider the interference from the other terrestrial networks. As has been stated, the beam radius of the multibeam satellite covers hundreds of kilometers, tens of times larger than the cell radius of terrestrial wireless communication networks. Some sensor nodes may be outside of the terrestrial communication networks (TCNs) and can transmit through the multibeam satellite, while some other TCNs inside the satellite beam will bring interference to the satellite. Thus, there is potential interference from TCNs in the return link of the IWSMSNs.

The work in [31] investigated the spectrum and power efficiency of an extremely high-frequency (EHF) satellite system, being integrated with terrestrial systems, and showed that the terrestrial systems caused interference for the satellite communication system. The work in [32] analyzed the Shannon-theoretic approximation to the Gaussian cellular channel. Using the framework of Wyner’s Gaussian cellular channel, [33] studied the performance of the return link for the multibeam satellite systems under Rician fading, but did not consider the terrestrial interference. The work in [34,35,36] analyzed the Ka band of cognitive satellite communications with incumbent terrestrial networks, which mainly considered the interference from satellite terminals to the terrestrial networks in the return link of the satellite, as the Ka band is predominantly a kine of sight (LOS) channel. Yet, [37,38,39,40,41] have performed research mainly concerning the forward link of the hybrid satellite terrestrial communication systems. Hence, this paper first investigates the performance of the integrated wireless sensor and multibeam satellite networks experiencing the Rician fading channel under the terrestrial interference.

The main contributions of this paper are summarized as follows.

- The performance of integrated wireless sensor and multibeam satellite networks experiencing the Rician fading under the terrestrial interference is firstly analyzed. The hybrid beam models are constructed by introducing the inter-beam interference factor *α* and intra-beam interference factor *β*. The relationships between the substitution *α* and the beam isolation parameter *ρ*, the substitution *β* and the terrestrial interference elevation angle *φ* are also analyzed.
- The optimal joint decoding capacity and linear minimum mean square error capacity approximations are derived by using the Haar approximation for the return link of IWSMSNs. The simulations show that the interference factor *α* and *β* contribute different effects to the capacity of different beam models, and the number of sinks also affects the performance of the optimal capacity and MMSE capacity differently. The capacity is decreased obviously by the Rician fading and is increased to the limit as the Rician factor increases to infinity, which will be turned to the Gauss channel. Meanwhile, the optimal capacity can be considered as the upper bound of the IWSMSNs.

The rest of the paper is organized as follows. Section 2 describes the IWSMSNs’ architecture and signal model, the hybrid beam models are also constructed; Section 3 uses the Haar approximation to derive the optimal capacity and MMSE capacity; Section 4 shows the summary of the simulation numerical results and especially analyzes the effects of *α*, *β* and *K* on the performance of the IWSMSNs; finally, conclusions are presented in Section 5.

Notations: Bold lowercase and uppercase letters represent the vectors and matrices. ${\left(\cdot \right)}^{†}$ and ${\left(\;\cdot \;\right)}^{H}$ respectively denote the Hermitian transpose and the transpose of vectors or matrices. ⊗ is the Kronecker product operator, and $E\left[\cdot \right]$ is the expectation operator. Tr(·) and det(·) represent the trace and determinant of a matrix. $R^{u\times v}$ and $C^{u\times v}$ stand for spaces of u×v real and complex matrices. The operators $\left|\cdot \right|$ and $∥\cdot ∥$ respectively denote the absolute value and two-norm of the Frobenius norm. $1_{u}=\left(1,...,1\right)$ is the 1×u all one vector, and $I_{u}$ is the u×u unit diagonal matrix. While ${}^{\circ }$ is the degree.

## 2. Network Architecture and Signal Model

### 2.1. Network Architecture

**Figure 1:**
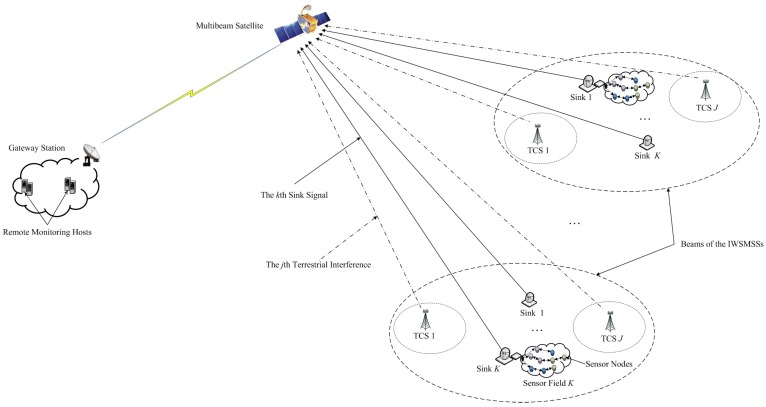
Integrated wireless sensor and multibeam satellite networks’ architecture.

Figure 1 shows the architecture of the integrated wireless sensor and multibeam satellite networks under the terrestrial interference. IWSMSNs are composed of sensor nodes (sensing), multibeam satellite (communication transmission) and remote monitoring hosts (data processing). The multibeam satellite covers multiple beams and multiple sensor fields connecting multiple satellite sinks per beam, while multiple TCNs may cause interference to the satellite. Obviously, multibeam satellite networks connect SNs to RMHs. The following provides more detail.

Sensor nodes: SNs sense different dynamic physical quantities for different scenarios, which can translate the quantities into electrical signals through the appropriate devices [20]. The electrical signals can be digitalized and encapsulated into data packets and transmitted to RMHs through multibeam satellite networks.

Multibeam satellite: The multibeam satellite can be considered as the important component of the IWSMSNs to connect SNs and RMHs. In Figure 1, we see that the sinks transmit the sensing data, including audio, video, images and other data streams, from the SNs to the gateway station, which exchanges the information with the RMHs through the return link of the satellite. The return link of multibeam a geosynchronous earth orbit (GEO) satellite is assumed to be operated at the S/L band where the channels experience the Rician fading [33,42]. The multibeam technology can improve the frequency and power efficiency, which reduces the required antenna aperture and power amplifier of sinks to lower the communication cost [42,43].

Remote monitoring hosts: RMHs mainly receive and process data packets from the SNs to obtain the required information.

Terrestrial interference: The terrestrial interferences are mostly from the sidelobe interferences of the adjacent channel or co-channel from the terrestrial communication networks.

### 2.2. Signal Model

**Figure 2:**
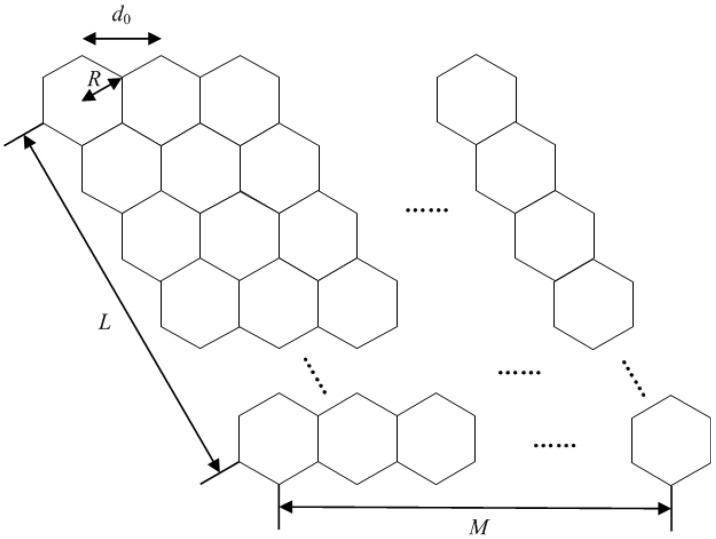
The hybrid 2D beam arrangement.

Figure 2 shows the 2D beam arrangement [33]. We define it as a hybrid 2D beam arrangement, which can be regarded as the arrangement of the multiple beams of a satellite in Figure 1. *L* and *M* denote the rows and columns of the 2D beam, respectively. The IWSMSN mainly considers a multibeam satellite equipped with *N* antennas (feeds) covering *N* beams, *K* sinks (*K* sensor fields) per beam and *J* TCNs contributing to the terrestrial interference. *K* sinks per beam and the total number of sinks result in NK transmitting signals to the satellite, while *N* antennas or feeds and a super-receiver on board the satellite are assumed to receive all of the signals that can be considered as single input multiple output (SIMO) networks. The RMHs receive the sensing data via the multibeam satellite. Due to the characteristics of the satellite channel, the satellite sinks are equipped with a single antenna. The sinks transmit signals (sensing data) to the satellite, while signals transmitted by TCNs are considered as the terrestrial interference to the satellite.

The return link of the satellite is regarded as cognitive and cooperative, which is due to the limits of satellite payloads. This paper assumes the perfect symmetry for the IWSMSN; meanwhile, the IWSMSN is also considered under the Rician fading and channel interference. *K* sinks per beam transmit signals to the satellite, and *J* terrestrial interferences also exist per beam. Thus, the antennas (feeds) on board the satellite receiving signals from sinks can be modeled as:

(1) y=HRs+n

where y is the received signal vector on board the satellite and $y\in C^{N\times 1}$. H is the channel matrix and $H\in C^{N\times NK}$, which includes the hybrid interference matrix T and will be discussed later. R is the fading matrix and $R\in C^{NK\times NK}$. s is the transmitted signal vector from *K* sinks in each beam, $s\in C^{NK\times 1}$, and $E[ss^{*}]=PI_{NK}$. n is the vector of complex circularly symmetric (c.c.s.) Gaussian noise with zero mean, covariance $E\left[nn^{†}\right]=\sigma^{2}I_{N}$ and $n\in C^{N\times 1}$.

The Rician model is usually adopted for the satellite communication networks as they are mainly under the direct LOS propagation channel. The Rician fading coefficient *κ* is independent and identically distributed (i.i.d.) c.c.s. Gaussian with independent real and imaginary parts, which is distributed as $N(\mu_{R}/\sqrt{2},\delta_{R}^{2})$ and can be defined as [44]:

(2) $$\kappa =\frac{\mu_{R}^{2}}{2\delta_{R}^{2}}$$

Moreover, the Rician fading can be converted to the nonfading channel as κ→∞, while the Rician fading can also be converted to the Rayleigh fading as κ=0. The signals transmitted from sinks are assumed to be subjected to the Rician fading, and the receiver on board the satellite or on board a gateway station knows the fading coefficient of each sink from the return link of the IWSMSNs.

The gain coefficient from *K* sinks in each beam to the satellite are assumed approximately equal, because the distances between sinks are about 1% compared with the distance from the sinks to the satellite, as the beam radius may range from 100–300 km, except in the extreme case of sinks distributed on the beam edge. This assumption could be seen in [33]. Then, the channel gain matrix can be given by:

(3) $$H=T⊗1_{K}$$

where T is defined as the interference matrix for the hybrid 1D and 2D beam models. Thus, the receiving signals under the fading channel can be rewritten as:

(4) $$y=(T⊗1_{K})Rs+n$$

### 2.3. Hybrid Interference Matrix

The interference matrix T is constructed from the following analysis. The antennas on board the satellite receive signals, including the desired signals from SSs intra-beam to be modeled as an equivalent factor 1, interference signals from TCNs intra-beam to be modeled as an equivalent interference factor *β* and interference signals from SSs inter-beam and TCNs inter-beam respectively to be modeled as equivalent interference factors *α* and *αβ*, respectively. *α* can be used as a substitute for $\alpha =10^{-4\rho }$, where *ρ* is defined as a parameter to vary beam isolation [32]. The *j*-th interference signal from the TCNs is assumed to be equivalent to $\beta_{j}$, and the interference from the TCNs per beam is equivalent to $\beta =\beta_{1}+...+\beta_{J}\;\left(\;1\leq j\leq J\right)$. From the Annex 4 of International Telecommunications Union-Radio (ITU-R) F.1336-4 [45], the mathematical model of omnidirectional generic average radiation patterns can be approximated and used for terrestrial antenna radiation patterns and are expressed as $\beta_{j}=G\left(\varphi \right)$. The following average side-lobe patterns should be used for elevation angles that range from −$90^{\circ }$ to $90^{\circ }$, given by [45]

(5) $$G\left(\varphi \right)=\left\{\begin{array}{cc} G_{0}-12{\left(\frac{\varphi }{\varphi_{3}}\right)}^{2}, & 0\leq \left|\varphi \right|<\varphi_{4}\\ G_{0}-12+10\log\left(t+1\right)+F\left(\varphi \right), & \varphi_{4}\leq \left|\varphi \right|<\varphi_{3}\\ G_{0}-12+10\log\left[{\left(\frac{\left|\varphi \right|}{\varphi_{3}}\right)}^{-1.5}+k\right]+F\left(\varphi \right), & \varphi_{3}\leq \left|\varphi \right|\leq 90^{\circ } \end{array}\right.$$

with:

$\varphi_{3}=107.6\times 10^{-0.1G_{0}}$
$\varphi_{4}=\varphi_{3}\sqrt{1-\frac{1}{1.2}\log\left(t+1\right)}$
$G_{0}=2+15\log\left(D/\lambda \right)$
$F\left(\varphi \right)=10\log\left(0.9\sin^{2}\left(\frac{3\pi \varphi }{4\varphi_{3}}\right)+0.1\right)$

where:

G(φ): gain relative to an isotropic antenna (dBi).
$G_{0}$: the maximum gain in the azimuth plane (dBi).
*φ*: elevation angle relative to the angle of the maximum gain (degrees) $\left(-90^{\circ }\leq \varphi \leq 90^{\circ }\right)$.
$\varphi_{3}$: the 3-dB beamwidth in the elevation plane (degrees).
*t*: in cases involving typical antennas operating in the 1–3 GHz range, the parameter *t* should be 0.7; in cases involving antennas with improved side-lobe performance in the 1–3 GHz range and for all antennas operating in the 3–70 GHz range, the parameter *t* should be zero.

**Figure 3:**
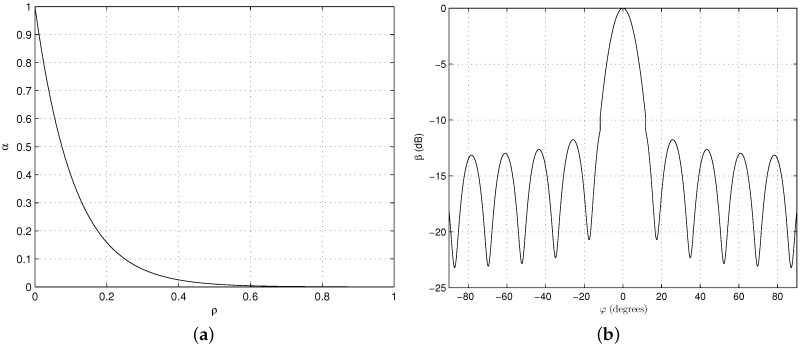
The relationships between the substitution and the practical parameter. (**a**) *α* versus beam isolation parameter *ρ*; (**b**) Normalized radiation pattern of *β* versus elevation angle *φ*.

Figure 3 shows the relationships between the substitution *α* and beam isolation parameter *ρ*, the substitution *β* and elevation angle *φ*. In Figure 3b, we can see that the terrestrial interference for the satellite depends on the terrestrial antenna elevation angle (λ= 0.1 m, D= 0.3 m, where *λ* is the wavelength and *D* is the equivalent antenna diameter); there may be tens of terrestrial communication networks per satellite beam. Whilst *α* and *β* may not reflect the actual characteristics of the beam patterns and antenna patterns, they can be utilized to analyze the effects of *α* and *β* on the performance of IWSMSNs.

The arrangement of a hybrid 1D beam is 1×N, where L=1 and M=N, as shown in Figure 2. The 1D beam interference matrix can be expressed as $T_{1D}$ [32], while only considering satellite sink interference from the adjacent beams:

(6) $$T_{1D}=\left[\begin{array}{cccc} 1 & \alpha & & \\ \alpha & 1 & \ddots & \\ & \ddots & \ddots & \alpha \\ & & \alpha & 1 \end{array}\right]$$

The 1D terrestrial interference matrix from the TCNs intra-beam and inter-beam can be expressed as $B_{1D}$, while again, only considering the TCNs interference from the adjacent beams:

(7) $$B_{1D}=\beta \left[\begin{array}{cccc} 1 & \alpha & & \\ \alpha & 1 & \ddots & \\ & \ddots & \ddots & \alpha \\ & & \alpha & 1 \end{array}\right]$$

Thus, the received signals intra-beam can be equivalent to (1+β), and the normalized desired signal can be equivalent to 11(1+β)(1+β). The interference from adjacent beams is equivalent to (α+αβ). The hybrid 1D interference matrix ${\bar{T}}_{1D}$ is an N×N Toeplitz block Toeplitz (TBT) matrix as:

(8) $${\bar{T}}_{1D}\;=\;\frac{1}{1\;+\;\beta }\;\left[\begin{array}{cccc} 1 & \alpha (1\;+\;\beta ) & & \\ \alpha (1\;+\;\beta ) & 1 & \ddots & \\ & \ddots & \ddots & \alpha (1\;+\;\beta )\\ & & \alpha (1\;+\;\beta ) & 1 \end{array}\right]\;$$

If the terrestrial interference and beam interference from all beams are considered, then the modified hybrid 1D interference matrix ${\hat{\bar{T}}}_{1D}$ is also constructed as an N×N TBT matrix:

(9) $${\hat{\bar{T}}}_{1D}=\left[\begin{array}{cccc} \frac{1}{\left(1+\beta \right)} & \alpha & \cdots & \alpha^{N-1}\\ \alpha & \frac{1}{\left(1+\beta \right)} & \cdots & \alpha^{N-2}\\ \vdots & \vdots & \ddots & \vdots \\ \alpha^{N-1} & \alpha^{N-2} & \cdots & \frac{1}{\left(1+\beta \right)} \end{array}\right]$$

The arrangement of a hybrid 2D beam is L×M, and the total beam number is N=L×M as shown in Figure 2, where *L* and *M* are respectively the rows and columns of the hybrid 2D beam. While the hybrid 2D interference matrix just considers the TCNs interference and beam interference from the adjacent beams, thus the hybrid 2D interference matrix ${\bar{T}}_{2D}$ can be constructed as a (L×M)×(L×M) TBT matrix, constituted by L×L submatrices, and the submatrix is M×M, given by:

(10) $${\bar{T}}_{2D}=\left[\begin{array}{cccc} {\bar{T}}_{1D} & \bar{S} & & \\ {\bar{S}}^{H} & {\bar{T}}_{1D} & \ddots & \\ & \ddots & \ddots & \bar{S}\\ & & {\bar{S}}^{H} & {\bar{T}}_{1D} \end{array}\right]$$

where ${\bar{T}}_{1D}$ is defined in Equation (8) and considers the interference from the adjacent beam of the same row in the 2D beam model. $\bar{S}$ considers the interference from the adjacent beam of the same column in the 2D beam model and can be constructed as:

(11) $$\bar{S}=\frac{1}{\left(1+\beta \right)}\left[\begin{array}{cccc} \alpha +\alpha \beta & & & \\ \alpha +\alpha \beta & \alpha +\alpha \beta & & \\ & \ddots & \ddots & \\ & & \alpha +\alpha \beta & \alpha +\alpha \beta \end{array}\right]$$

Then, we obtain the eigenvalues of the hybrid matrix, which will be used in the capacity approximation. The eigenvalues of ${\bar{T}}_{1D}$ for the hybrid 1D model are derived as [46]:

(12) $$\lambda_{i}\left({\bar{T}}_{1D}\right)=\frac{1}{1+\beta }\left(1+2\alpha \left(1+\beta \right)\cos\left(\frac{i\pi }{N+1}\right)\right).\;i=1,...,N.$$

The eigenvalues of ${\hat{\bar{T}}}_{1D}$ for the modified hybrid 1D model are derived as [46]:

(13) $$\lambda_{i}\left({\hat{\bar{T}}}_{1D}\right)=\left(\frac{1}{1+\beta }-1\right)+\frac{1-\alpha^{2}}{1+2\alpha \cos\left(\frac{i\pi +\varepsilon }{N+1}\right)+\alpha^{2}}$$

The eigenvalues of ${\bar{T}}_{2D}$ for the hybrid 2D model are derived as [47]:

(14) λ(l-1)M+m(T¯2D)=11+β(1+2α(1+β)(cos(2πl2πlLL)+cos(2πm2πmMM)+cos(2πl2πlLL+2πm2πmMM))).l=1,...,L,m=1,...,M.

## 3. Capacity Analysis of the IWSMSNs

The multibeam satellite has previously been stated as operated at the S/L band, and the Rician fading channel is used as the LOS return link of IWSMSNs. The optimal joint decoding capacity and linear MMSE capacity are analyzed under the Rician fading channel. The Haar measure approximations are utilized to derive the closed form approximations of the optimal capacity and MMSE capacity; [48] has proven that the random Haar matrices could get the asymptotic performance as most deterministic precoding matrices.

### 3.1. The Optimal Joint Decoding Capacity

$r_{n,k}$ is denoted as the Rician fading coefficient for the *k*-th sink in the *n*-th beam, and R = diag$\left(r_{1,1},...,r_{1,K},...,r_{N,1},...,r_{N,K}\right)$. The Rician fading coefficient $r_{n,k}$ is assumed to be complex, independent, strictly stationary and ergodic and normalized as $E[r_{n,k}r_{n,k}^{*}]=1$. Because the distance between the sink and receiving antennas (feeds) on board the satellite is far longer than the distance between the receiving antennas (feeds) on board the satellite, thus the signal of the sink to the satellite would experience the same fading at every receiving antenna on board the satellite. It is quite clear that the same fading to the receiving antenna is different from the TCNs where each sink experiences independent fading at every receiving antenna of the base stations. Sinks from all beams can be optimally jointly decoded by a super-receiver on-board the gateway station or on-board the satellite to be as the optimal joint decoding. The optimal capacity per beam can be expressed as the log-determinant formula [49]:

(15) $$\begin{array}{c} \lim_{N\to \infty }C_{\mathrm{opt}}\left(HR;\alpha ,\beta ,\gamma \right)\\ =\lim_{N\to \infty }C_{\mathrm{opt}}\left(\left(T⊗1_{K}\right)R;\alpha ,\beta ,\gamma \right)\\ =\frac{1}{N}E\left[\mathrm{logdet}(I_{N}+\gamma \left(T⊗1_{K}\right)RR^{†}{\left(T⊗1_{K}\right)}^{†}\right]\\ =\frac{1}{N}E\left[\mathrm{logdet}(I_{N}+\gamma T\Theta T^{†}\right]\\ =\frac{1}{N}E\left[\mathrm{logdet}(I_{N}+\gamma T^{†}T\Theta \right] \end{array}$$

where $\Theta =\mathrm{diag}\left\{\theta_{1},...,\theta_{N}\right\}\in C^{N\times N}$, and $\theta_{n}=\sum_{k=1}^{K}(r_{n,k}r_{n,k}^{*})$. $\gamma =P/\sigma^{2}$ is the signal-to-noise ratio (SNR) for the receiver, while the interference and Rician fading will be analyzed in the matrices H and R. From Equations (6)–(8), the interference matrix T can be considered as a fixed matrix for *N*; *α* and *β* are constant as fixed. The fading matrix R is distributed as i.i.d. c.c.s. Gaussian with independent real and imaginary parts. T and R generally are not unitary matrices. The work in [48] has shown that the Haar distributed approximations achieved the asymptotic performance with the theoretical performance. Thus, we adopt the Haar distributed approximations to solve the problem, where the Haar matrix is employed to replace the fixed matrix T. The capacity approximations will converge as *N* increases. $T^{†}T$ can be decomposed as $T^{†}T=Q\Sigma Q^{†}$, where Q is a unitary matrix and **Σ** is a diagonal matrix. A Haar matrix W is used to replace the fixed matrix and $T^{†}T\approx W\Sigma W^{†}$ [50] and $T^{†}T\Theta \approx W\Sigma W^{†}\Theta =\Sigma W\Theta W^{†}$. The approximation will be more precisely asymptotic as the beam number *N* increases. Thus, the Haar approximation of capacity is given by:

(16) $$\begin{array}{c} \lim_{N\to \infty }C_{\mathrm{OPT}}\left(\left(T⊗1_{K}\right)R;\alpha ,\beta ,\gamma \right)\\ \approx \frac{1}{N}E\left[\mathrm{logdet}(I_{N}+\gamma \Sigma W\Theta W^{†})\right] \end{array}$$

From [51], the moment-generating function (MGF) $F_{MGF}\left(u\right)$ of f(X) is given by E[exp(u·f(X))]. In order to get the approximations of $C_{\mathrm{OPT}}$, therefore, the MGF of $\mathrm{logdet}(I_{N}+\gamma \Sigma W\Theta W^{†})$ can be rewritten as:

(17) $$\begin{array}{c} F\left(u\right)\approx E\left[\exp\left(u\cdot \mathrm{logdet}(I_{N}+\gamma \Sigma W\Theta W^{†})\right)\right]\\ =E\left[\det\left(I_{N}+\gamma \Sigma W\Theta W^{†}\right){}^{u}\right]\\ \approx \int_{W}\det\left(I_{N}+\gamma \Sigma W\Theta W^{†}\right){}^{u}dW \end{array}$$

dW is defined as the Haar measure from [52]. In [53], the integral can be written as a hypergeometric function and expressed in terms of a scalar argument,

(18) $$\begin{array}{c} F\left(u\right)=\int_{W}\det\left(I_{N}+\gamma \Sigma W\Theta W^{†}\right){}^{u}dW\\ {=}_{1}F_{0}\left(-u;-\gamma \Sigma ,\Theta \right)\\ =\frac{\prod_{n=1}^{N}(n-1)!}{\prod_{n=1}^{N}{\left(-u-N+1\right)}_{N-n}}\cdot \frac{\det\left({}_{1}F_{0}\left(-u-N+1;-\gamma \zeta_{i}\theta_{j}\right)\right)}{V(-\gamma \Sigma )V(\Theta )}\\ =\frac{\prod_{n=1}^{N}(n-1)!}{\prod_{n=1}^{N}{\left(-u-N+1\right)}_{N-n}}\cdot \frac{\det\left({\left(1+\gamma \theta_{i}\zeta_{j}\right)}^{u+N-1}\right){}_{i,j}}{V(-\gamma \Sigma )V(\Theta )} \end{array}$$

where V(X) denotes the Vandermonde determinant and $\lambda_{s}$ are the eigenvalues of X. $V\left(X\right)=\prod_{s>t}\left(\lambda_{s}-\lambda_{t}\right)$, and $V\left(bX\right)=b^{\sum_{n=1}^{N}n}V\left(X\right)$. $\zeta_{i}$ and $\theta_{j}$ are the diagonal element of diagonal matrices **Σ** and **Θ**, respectively. Meanwhile, ${}_{1}F_{0}\left(a,x\right)={\left(1-x\right)}^{-a}$ [54]. ${\left(\right)}_{i,j}$ expresses the element of the *i*-th row and *j*-th column for a matrix, and we denote $Y\left(u\right)={\left\{{\left(1+\gamma \theta_{i}\zeta_{j}\right)}^{u+N-1}\right\}}_{i,j}$. From [51], the mathematical expectation [exp(u·f(X))] can be obtained by $E\left[X\cdot \exp\left(u\cdot f\left(X\right)\right)\right]=\frac{dF_{MGF}\left(u\right)}{du}$, and $E\left[X\right]={\left.\frac{dF_{MGF}\left(u\right)}{du}\right|}_{u=0}$; then:

(19) $$\lim_{N\to \infty }C_{\mathrm{OPT}}\left(\left(T⊗1_{K}\right)R;\alpha ,\beta ,\gamma \right)=\frac{1}{N}{\left.\frac{dF(u)}{du}\right|}_{u=0}$$

Hence, we must derive $\frac{dF(u)}{du}$:

(20) $$\begin{array}{c} \frac{dF(u)}{du}=\frac{d\left(\frac{\prod_{n=1}^{N}(n-1)!}{V(-\gamma \Sigma )V(\Theta )}\cdot \frac{\det Y(u)}{\prod_{n=1}^{N}{\left(-u-N+1\right)}_{N-n}}\right)}{du}\\ =\frac{\prod_{n=1}^{N}(n-1)!}{V(-\gamma \Sigma )V(\Theta )}\cdot \frac{d\left(\frac{\det Y(u)}{\prod_{n=1}^{N}{\left(-u-N+1\right)}_{N-n}}\right)}{du} \end{array}$$

Then, we denote $G\left(u\right)=\prod_{n=1}^{N}{\left(-u-N+1\right)}_{N-n}$, and use the quotient rule:

(21) $$\begin{array}{c} \frac{d\left(\frac{\det Y(u)}{\prod_{n=1}^{N}{\left(-u-N+1\right)}_{N-n}}\right)}{du}\\ =\frac{d\left(\frac{\det Y(u)}{G(u)}\right)}{du}\\ =\frac{\frac{d(\det Y(u))}{du}\cdot G\left(u\right)-\det Y\left(u\right)\cdot \frac{d(G(u))}{du}}{G^{2}\left(u\right)} \end{array}$$

We must obtain $\frac{d\left(\det Y(u)\right)}{du}$ and $\frac{d\left(G(u)\right)}{du}$ to solve (21). From [55], the differential of determinant $d\left(\det F\left(X\right)\right)=\det F\left(X\right)\cdot \mathrm{tr}\left(F{\left(X\right)}^{-1}dF\left(X\right)\right)$; thus, we get:

(22) $$\begin{array}{c} \frac{d\left(\det Y(u)\right)}{du}=\det Y\left(u\right)\cdot \mathrm{tr}\left(Y{\left(u\right)}^{-1}\cdot \frac{dY(u)}{du}\right)\\ =\det Y\left(u\right)\cdot \mathrm{tr}\left({\left({\left({\left(1+\gamma \theta_{i}\zeta_{j}\right)}^{u+N-1}\right)}_{i,j}\right)}^{-1}\cdot \frac{d{\left({\left(1+\gamma \theta_{i}\zeta_{j}\right)}^{u+N-1}\right)}_{i,j}}{du}\right)\\ =\det Y\left(u\right)\cdot \mathrm{tr}\left({\left({\left({\left(1+\gamma \theta_{i}\zeta_{j}\right)}^{u+N-1}\right)}_{i,j}\right)}^{-1}\cdot {\left(\log\left(1+\gamma \theta_{i}\zeta_{j}\right)\cdot {\left(1+\gamma \theta_{i}\zeta_{j}\right)}^{u+N-1}\right)}_{i,j}\right)\\ =\det Y\left(u\right)\cdot \mathrm{tr}\left(Z\left(u\right)\cdot {\left(\log\left(1+\gamma \theta_{i}\zeta_{j}\right)\cdot {\left(1+\gamma \theta_{i}\zeta_{j}\right)}^{u+N-1}\right)}_{i,j}\right) \end{array}$$

where Z(u) is defined as $Z\left(u\right)={\left({\left({\left(1+\gamma \theta_{i}\zeta_{j}\right)}^{u+N-1}\right)}_{i,j}\right)}^{-1}$. As we want to solve $\frac{d\left(G(u)\right)}{du}$, we rearrange the factorial order and get:

(23) $$\begin{array}{c} \prod_{n=1}^{N}{\left(-u-N+1\right)}_{N-n}\\ ={\left(-u-N+1\right)}_{N-1}{\left(-u-N+1\right)}_{N-2}\ldots {\left(-u-N+1\right)}_{1}\left(-u-N+1\right)\\ ={\left(-u-N+1\right)}^{N-1}{\left(-u-N+2\right)}^{N-2}\ldots {\left(-u-2\right)}^{1}\left(-u-1\right)\\ =\prod_{n=1}^{N-1}{\left(-u-n\right)}^{n} \end{array}$$

Since $\log\left(\prod_{n=1}^{N-1}{\left(-u-n\right)}^{n}\right)=\sum_{n=1}^{N-1}n\log(-u-n)$, then:

(24) $$\begin{array}{c} \frac{d\log\left(G(u)\right)}{du}=\frac{d\left(G(u)\right)}{du}\frac{1}{G(u)}\\ =\frac{d\left(\sum_{n=1}^{N-1}n\log(-u-n)\right)}{du}\\ =\sum_{n=1}^{N-1}n\frac{d\log(-u-n)}{du}\\ =\sum_{n=1}^{N-1}\frac{n}{u+n} \end{array}$$

Then, we can get that:

(25) $$\frac{d\left(G(u)\right)}{du}=G\left(u\right)\sum_{n=1}^{N-1}\frac{n}{u+n}$$

We substitute the Equations (22) and (25) into Equation (21), then:

(26) $$\begin{array}{c} \frac{\frac{d\det Y(u)}{du}\cdot G\left(u\right)-\det Y\left(u\right)\cdot \frac{dG(u)}{du}}{G^{2}\left(u\right)}\\ =\frac{\det Y\left(u\right)\cdot \mathrm{tr}\left({\left({\left({\left(1+\gamma \theta_{i}\zeta_{j}\right)}^{u+N-1}\right)}_{i,j}\right)}^{-1}\cdot {\left(\log\left(1+\gamma \theta_{i}\zeta_{j}\right)\cdot {\left(1+\gamma \theta_{i}\zeta_{j}\right)}^{u+N-1}\right)}_{i,j}\right)\cdot G\left(u\right)-\det Y\left(u\right)\cdot G\left(u\right)\sum_{n=1}^{N-1}\frac{n}{u+n}}{G^{2}\left(u\right)}\\ =\frac{\det Y(u)}{G(u)}\left(\mathrm{tr}\left(Z\left(u\right)\cdot {\left(\log\left(1+\gamma \theta_{i}\zeta_{j}\right)\cdot {\left(1+\gamma \theta_{i}\zeta_{j}\right)}^{u+N-1}\right)}_{i,j}\right)-\sum_{n=1}^{N-1}\frac{n}{u+n}\right) \end{array}$$

After substituting Equations (20) and (26) into Equation (19), we derive the optimal capacity of the return link for the IWSMSNs as:

(27) $$\begin{array}{c} \lim_{N\to \infty }C_{\mathrm{OPT}}\left(\left(T⊗1_{K}\right)R;\alpha ,\beta ,\gamma \right)\\ \approx \frac{1}{N}\frac{\prod_{n=1}^{N}(k-1)!}{V(-\gamma \Sigma )V(\Theta )}\frac{\det Y(u)}{G(u)}\;\cdot \;{\left.\left(\mathrm{tr}\left(Z\left(u\right)\cdot {\left(\log\left(1+\gamma \theta_{i}\zeta_{j}\right)\cdot {\left(1+\gamma \theta_{i}\zeta_{j}\right)}^{u+N-1}\right)}_{i,j}\right)\;-\;\;\sum_{n=1}^{N-1}\;\frac{n}{u+n}\right)\right|}_{u=0} \end{array}$$

To compute the optimal capacity approximation (27), $\theta_{i}$ can be determined as $\theta_{i}\approx \lambda_{i}^{2}\left(T\right)$, and $\lambda_{i}\left(T\right)$ is approximately expressed as Equations (12) and (13) respectively for the hybrid 1D beam model and modified hybrid 1D beam model. Since $\lambda_{(l-1)M+m}\left({\bar{T}}_{2D}\right)$ of the hybrid 2D beam model are affected by *L* and *M* and computed repeatedly in (27), we use the integral of the inverse function [51] to get $\theta_{i}$ for the hybrid 2D beam model,

(28) $$F_{{\bar{T}}_{2D}}\left(x\right)=\frac{1}{\pi }\int_{0}^{1}\arccos\left(\frac{1}{2\cos0.5\pi t}\left(\frac{x-\frac{1}{1+\beta }}{2\alpha }-\cos\pi t\right)\right)dt$$

which can be computed as $F_{{\bar{T}}_{2D}^{2}}^{-1}\left(x\right)$. The distribution of *x* was determined by $\lambda_{(l-1)M+m}\left({\bar{T}}_{2D}\right)$ and by using the standard probability theory [51].

For the Rician fading channel, $f_{\zeta }\left(x\right)$ is a noncentral chi-squared density [56],

(29) $$f_{\zeta }\left(x\right)=\left(\kappa +1\right){\left(\frac{(\kappa +1)x}{K\kappa }\right)}^{\frac{K-1}{2}}e^{-((\kappa +1)x+K\kappa )}I_{K-1}\left(2\sqrt{K\kappa (\kappa +1)x}\right)$$

where $I_{n}\left(x\right)$ is the first kind of modified Bessel function and the order is *n*. Thus, $\zeta_{j}$ can be computed as $\zeta_{j}\approx F_{\zeta^{2}}\left(x\right)$.

### 3.2. The Linear MMSE Capacity

Another important performance for the capacity of IWSMSNs is the linear MMSE. The work in [57] proposed a framework to describe the linear MMSE performance of the return link for the multibeam satellite communication systems, which analyzed average MMSE spectral efficiency per user of the multibeam satellite systems and did not give a closed approximation for the MMSE capacity. The work in [58] investigated the impact of spatially-correlated rain attenuation on the performance of a multibeam satellite return link above the K-band. The work in [58] also did not give the closed approximations of MMSE capacity for the multibeam satellite systems under Rician fading. Thus, the arithmetic mean over the *K* sinks per beam of the MMSE is given by [49]:

(30) $$\begin{array}{c} \mathrm{MMSE}{}_{avg}\left(\left(T⊗1_{K}\right)R;\alpha ,\beta ,\gamma \right)\\ =\frac{1}{NK}\min E\left[{∥x-My∥}^{2}\right]\\ =\frac{P}{NK}\mathrm{tr}\left\{{\left(I_{N}+K\gamma T\Theta T^{†}\right)}^{-1}\right\}\\ =P\left(1-\frac{1}{NK}\gamma \frac{d}{d\gamma }\left(\mathrm{logdet}(I_{N}+K\gamma T\Theta T^{†})\right)\right) \end{array}$$

where M is the linear MMSE filter and My is the linear MMSE filter output. The linear MMSE capacity has been studied in [49], and we also adopt the Haar approximation to obtain the MMSE capacity, given by:

(31) $$\begin{array}{c} \lim_{N\to \infty }C_{\mathrm{MMSE}}\left(\left(T⊗1_{K}\right)R;\alpha ,\beta ,\gamma \right)\\ =K\log(\frac{P}{\mathrm{MMSE}{}_{avg}\left(\left(T⊗1_{K}\right)R;\alpha ,\beta ,\gamma \right)})\\ \approx -K\log\left(1-\frac{1}{NK}\gamma \frac{d}{d\gamma }\left(\mathrm{logdet}(I_{N}+K\gamma T\Theta T^{†})\right)\right) \end{array}$$

From Equation (19), we have $\mathrm{logdet}\left(I_{N}+K\gamma T\Theta T^{†}\right)\approx {\left.\frac{dF(u)}{du}\right|}_{u=0}$; thus:

(32) $$\begin{array}{c} \lim_{N\to \infty }C_{\mathrm{MMSE}}\left(\left(T⊗1_{K}\right)R;\alpha ,\beta ,\gamma \right)\\ \approx -{\left.K\log\left(1-\frac{1}{NK}\gamma \frac{d}{d\gamma }\left(\frac{dF(u)}{du}\right)\right)\right|}_{u=0} \end{array}$$

In order to solve $C_{\mathrm{MMSE}}$, we must derive $\frac{d}{d\gamma }\left(\frac{dF(u)}{du}\right)$:

(33) $$\begin{array}{c} \frac{d}{d\gamma }\left(\frac{dF(u)}{du}\right)=\frac{d}{d\gamma }\left(\frac{1}{N}\frac{\prod_{n=1}^{N}(k-1)!}{V(-\gamma \Sigma )V(\Theta )}\frac{\det Y(u)}{G(u)}\;\cdot \;\left(\mathrm{tr}\left(\;Z\left(u\right)\cdot \;{\left(\log\left(1+\gamma \theta_{i}\zeta_{j}\right)\cdot {\left(1+\gamma \theta_{i}\zeta_{j}\right)}^{u+N-1}\right)}_{i,j}\right)\;-\;\;\sum_{n=1}^{N-1}\frac{n}{u+n}\;\right)\right)\\ =\frac{1}{N}\frac{\prod_{n=1}^{N}(k-1)!}{V(\Sigma )V(\Theta )G(u)}\frac{d}{d\gamma }\left(\;{\left(-\gamma \right)}^{-\frac{N(N-1)}{2}}\;\;\cdot \;\det Y\left(u\right)\;\cdot \;\left(\mathrm{tr}\left(\;Z\left(u\right)\cdot \;{\left(\log\left(1+\gamma \theta_{i}\zeta_{j}\right)\cdot {\left(1+\gamma \theta_{i}\zeta_{j}\right)}^{u+N-1}\right)}_{i,j}\right)\;-\;\;\sum_{n=1}^{N-1}\frac{n}{u+n}\;\right)\right)\\ =\frac{1}{N}\frac{{\left(-\gamma \right)}^{-\frac{1}{2}N\left(N-1\right)}\prod_{n=1}^{N}(k-1)!}{V(\Sigma )V(\Theta )G(u)}\left(-\frac{1}{2\gamma }N\left(N-1\right)\;\cdot \;\det Y\left(u\right)\;\cdot \;\mathrm{tr}\left(\;Z\left(u\right)\cdot \;{\left(\log\left(1+\gamma \theta_{i}\zeta_{j}\right)\cdot {\left(1+\gamma \theta_{i}\zeta_{j}\right)}^{u+N-1}\right)}_{i,j}\right)\right.\\ +\frac{d}{d\gamma }\left(\det Y(u)\right)\cdot \mathrm{tr}\left(\;Z\left(u\right)\cdot \;{\left(\log\left(1+\gamma \theta_{i}\zeta_{j}\right)\cdot {\left(1+\gamma \theta_{i}\zeta_{j}\right)}^{u+N-1}\right)}_{i,j}\right)\\ +\det Y\left(u\right)\cdot \frac{d}{d\gamma }\left(\mathrm{tr}\left(\;Z\left(u\right)\cdot \;{\left(\log\left(1+\gamma \theta_{i}\zeta_{j}\right)\cdot {\left(1+\gamma \theta_{i}\zeta_{j}\right)}^{u+N-1}\right)}_{i,j}\right)\right)\\ \left.+\frac{1}{2\gamma }N\left(N-1\right)\cdot \det Y\left(u\right)\cdot \sum_{n=1}^{N-1}\frac{n}{u+n}-\frac{d}{d\gamma }\left(\det Y(u)\right)\cdot \sum_{n=1}^{N-1}\frac{n}{u+n}\right) \end{array}$$

Being similar to the process of solving Equation (22), we get:

(34) $$\begin{array}{c} \frac{d}{d\gamma }\left(\det Y(u)\right)\\ =\det Y\left(u\right)\cdot \mathrm{tr}\left(Y{\left(u\right)}^{-1}\cdot \frac{dY(u)}{d\gamma }\right)\\ =\det Y\left(u\right)\cdot \mathrm{tr}\left({\left({\left({\left(1+\gamma \theta_{i}\zeta_{j}\right)}^{u+N-1}\right)}_{i,j}\right)}^{-1}\cdot {\left(\frac{\left(u+N-1\right)\theta_{i}\zeta_{j}}{1+\gamma \theta_{i}\zeta_{j}}\cdot {\left(1+\gamma \theta_{i}\zeta_{j}\right)}^{u+N-1}\right)}_{i,j}\right)\\ =\det Y\left(u\right)\cdot \mathrm{tr}\left(Z\left(u\right)\cdot {\left(\frac{\left(u+N-1\right)\theta_{i}\zeta_{j}}{1+\gamma \theta_{i}\zeta_{j}}\cdot {\left(1+\gamma \theta_{i}\zeta_{j}\right)}^{u+N-1}\right)}_{i,j}\right) \end{array}$$

From [55], the differential of trace d(tr(X))=tr(d(X)); thus:

(35) $$\begin{array}{c} \frac{d}{d\gamma }\mathrm{tr}\left(Z\left(u\right)\cdot {\left(\log\left(1+\gamma \theta_{i}\zeta_{j}\right)\cdot {\left(1+\gamma \theta_{i}\zeta_{j}\right)}^{u+N-1}\right)}_{i,j}\right)\\ =\mathrm{tr}\left(\frac{d}{d\gamma }\left({\left({\left({\left(1+\gamma \theta_{i}\zeta_{j}\right)}^{u+N-1}\right)}_{i,j}\right)}^{-1}\cdot {\left(\log\left(1+\gamma \theta_{i}\zeta_{j}\right)\cdot {\left(1+\gamma \theta_{i}\zeta_{j}\right)}^{u+N-1}\right)}_{i,j}\right)\right)\\ =\mathrm{tr}\left(Z\left(u\right)\cdot {\left(\log\left(1+\gamma \theta_{i}\zeta_{j}\right)\cdot \frac{\left(u+N-1\right)\theta_{i}\zeta_{j}}{1+\gamma \theta_{i}\zeta_{j}}\cdot {\left(1+\gamma \theta_{i}\zeta_{j}\right)}^{u+N-1}\right)}_{i,j}\right.\\ +Z\left(u\right)\cdot {\left(\frac{\theta_{i}\zeta_{j}}{1+\gamma \theta_{i}\zeta_{j}}\cdot {\left(1+\gamma \theta_{i}\zeta_{j}\right)}^{u+N-1}\right)}_{i,j}\\ \left.-\frac{d}{d\gamma }{\left({\left({\left(1+\gamma \theta_{i}\zeta_{j}\right)}^{u+N-1}\right)}_{i,j}\right)}^{-1}\cdot {\left(\log\left(1+\gamma \theta_{i}\zeta_{j}\right)\cdot {\left(1+\gamma \theta_{i}\zeta_{j}\right)}^{u+N-1}\right)}_{i,j}\right) \end{array}$$

Furthermore, from [55], the differential of inverse matrix $d\left(X^{-1}\right)=-X^{-1}\left(dX\right)X^{-1}$; then, we get:

(36) $$\begin{array}{c} \frac{d\left(Z(u)\right)}{d\gamma }\\ =-{\left({\left({\left(1+\gamma \theta_{i}\zeta_{j}\right)}^{u+N-1}\right)}_{i,j}\right)}^{-1}\cdot \frac{d}{d\gamma }{\left({\left(1+\gamma \theta_{i}\zeta_{j}\right)}^{u+N-1}\right)}_{i,j}\cdot {\left({\left({\left(1+\gamma \theta_{i}\zeta_{j}\right)}^{u+N-1}\right)}_{i,j}\right)}^{-1}\\ =-Z\left(u\right)\cdot {\left(\frac{\theta_{i}\zeta_{j}}{1+\gamma \theta_{i}\zeta_{j}}\cdot {\left(1+\gamma \theta_{i}\zeta_{j}\right)}^{u+N-1}\right)}_{i,j}\cdot Z\left(u\right) \end{array}$$

After substituting Equations (32)–(36) into Equation (31), we derive the linear MMSE capacity of the return link for the IWSMSNs as:

(37) $$\begin{array}{c} \lim_{N\to \infty }C_{\mathrm{MMSE}}\left(\left(T⊗1_{K}\right)R;\alpha ,\beta ,\gamma \right)\\ \approx -K\log\left(1-\frac{\gamma }{N^{2}K}\right.\frac{{\left(-\gamma \right)}^{-\frac{1}{2}N\left(N-1\right)}\prod_{n=1}^{N}(k-1)!}{V(\Sigma )V(\Theta )G(u)}\\ \;\;\cdot \left(-\frac{1}{2\gamma }N\left(N-1\right)\cdot \det Y\left(u\right)\cdot \mathrm{tr}\left(Z\left(u\right)\cdot {\left(\log\left(1+\gamma \theta_{i}\zeta_{j}\right)\cdot {\left(1+\gamma \theta_{i}\zeta_{j}\right)}^{u+N-1}\right)}_{i,j}\right)\right.\\ \;\;+\det Y\left(u\right)\cdot \mathrm{tr}\left(Z\left(u\right)\cdot {\left(\frac{\left(u+N-1\right)\theta_{i}\zeta_{j}}{1+\gamma \theta_{i}\zeta_{j}}\cdot {\left(1+\gamma \theta_{i}\zeta_{j}\right)}^{u+N-1}\right)}_{i,j}\right)\\ \;\;\;\;\cdot \mathrm{tr}\left(Z\left(u\right)\cdot {\left(\log\left(1+\gamma \theta_{i}\zeta_{j}\right)\cdot {\left(1+\gamma \theta_{i}\zeta_{j}\right)}^{u+N-1}\right)}_{i,j}\right)\\ \;\;+\det Y\left(u\right)\cdot \mathrm{tr}\left(Z\left(u\right)\cdot {\left(\log\left(1+\gamma \theta_{i}\zeta_{j}\right)\cdot \frac{\left(u+N-1\right)\theta_{i}\zeta_{j}}{1+\gamma \theta_{i}\zeta_{j}}\cdot {\left(1+\gamma \theta_{i}\zeta_{j}\right)}^{u+N-1}\right)}_{i,j}\right.\\ \;\;\;\;+Z\left(u\right)\cdot {\left(\frac{\theta_{i}\zeta_{j}}{1+\gamma \theta_{i}\zeta_{j}}\cdot {\left(1+\gamma \theta_{i}\zeta_{j}\right)}^{u+N-1}\right)}_{i,j}\\ \;\;\;\;\left.+Z\left(u\right)\cdot {\left(\frac{\theta_{i}\zeta_{j}}{1+\gamma \theta_{i}\zeta_{j}}{\left(1+\gamma \theta_{i}\zeta_{j}\right)}^{u+N-1}\right)}_{i,j}\cdot Z\left(u\right)\cdot {\left(\log\left(1+\gamma \theta_{i}\zeta_{j}\right)\cdot {\left(1+\gamma \theta_{i}\zeta_{j}\right)}^{u+N-1}\right)}_{i,j}\right)\\ \;\;+\frac{1}{2\gamma }N\left(N-1\right)\cdot \det Y\left(u\right)\cdot \sum_{n=1}^{N-1}\frac{n}{u+n}\\ \;\;{\left.\left.-\det Y\left(u\right)\cdot \mathrm{tr}\left(Z\left(u\right)\cdot {\left(\frac{\left(u+N-1\right)\theta_{i}\zeta_{j}}{1+\gamma \theta_{i}\zeta_{j}}\cdot {\left(1+\gamma \theta_{i}\zeta_{j}\right)}^{u+N-1}\right)}_{i,j}\right)\cdot \sum_{n=1}^{N-1}\frac{n}{u+n}\right)\right|}_{u=0} \end{array}$$

To compute the MMSE capacity approximation (37), $\theta_{i}$ can also be determined as $\theta_{i}\approx \lambda_{i}^{2}\left(T\right)$. $\lambda_{i}\left(T\right)$ is approximately expressed as Equations (12) and (13) respectively for the hybrid 1D beam model and modified hybrid 1D beam model, and $\theta_{i}$ of the hybrid 2D beam model can be computed as $F_{{\bar{T}}_{2D}^{2}}^{-1}\left(x\right)$ in (28). Meanwhile, $\zeta_{j}$ can be determined as $\zeta_{j}\approx F_{\zeta^{2}}\left(x\right)$ in (29).

## 4. Simulations and Results

This section provides the numerical results to verify the performance of the integrated wireless sensor and multibeam satellite networks under terrestrial interference. A GEO multibeam satellite with *N* antennas or feeds is considered. The satellite covers *N* beams where N=1×N beams for the 1D beam model and N=L×M beams for the 2D beam model. There are *K* sinks with a single antenna as the sensor nodes or connecting the sensor fields, which can sense information in each beam, and *J* terrestrial interferences in each beam. *K* sinks in each beam transmit signals to the satellite, and *N* antennas or feeds and a super-receiver on board the satellite are assumed to receive all of the signals, which can be considered as SIMO networks. The L/S band is assumed for the return link, and the signals experience Rician fading. Either the satellite decodes the received signals or the gateway station decodes the signals, and then, the gateway station sends sensing information to the remote monitoring hosts. This paper mainly investigates the optimal capacity and linear MMSE capacity for the IWSMSNs under Rician fading. *α* is defined as the equivalent inter-beam interference from inter-beam and 0≤α≤1, while *β* is defined as the equivalent intra-beam interference from TCNs and 0≤β≤1. We mainly investigate the effects of *α* and *β* on the capacity. The numerical simulations are plotted by using MATLAB. In the simulations, the number of hybrid 1D beams is *N* = 300; the number of hybrid 2D beams is *L* = 50 and *M* = 50; and the Rician fading factor is chosen as *κ* = 0, *κ* = 10 and *κ* = 50.

**Figure 4:**
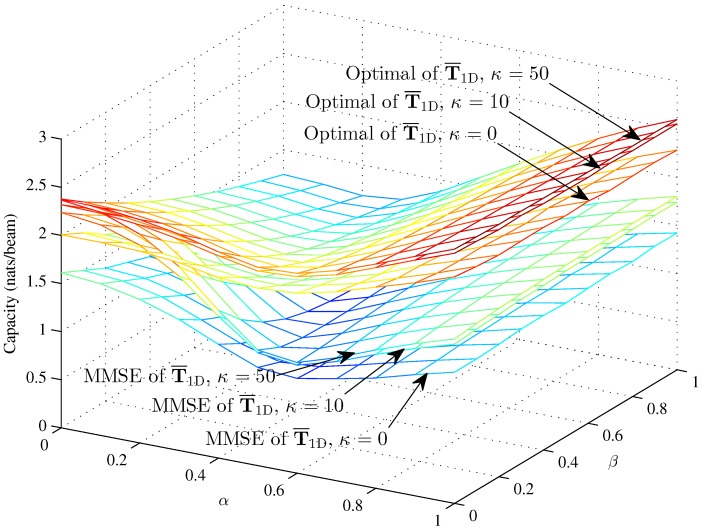
The optimal capacity and MMSE capacity of the hybrid 1D beams with *γ* = 10 and *N* = 100 (*κ* = 0, *κ* = 10 and *κ* = 50) versus *α* and *β*.

**Figure 5:**
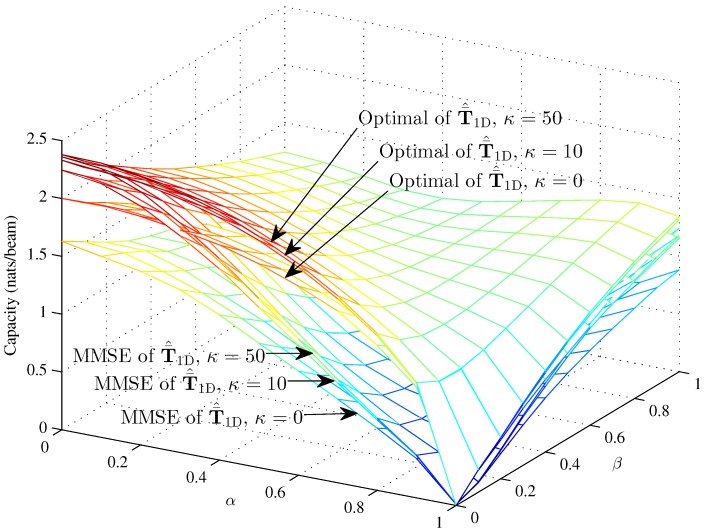
The optimal capacity and MMSE capacity of the modified hybrid 1D beams with *γ* = 10 and *N* = 100 (*κ* = 0, *κ* = 10 and *κ* = 50) versus *α* and *β*.

**Figure 6:**
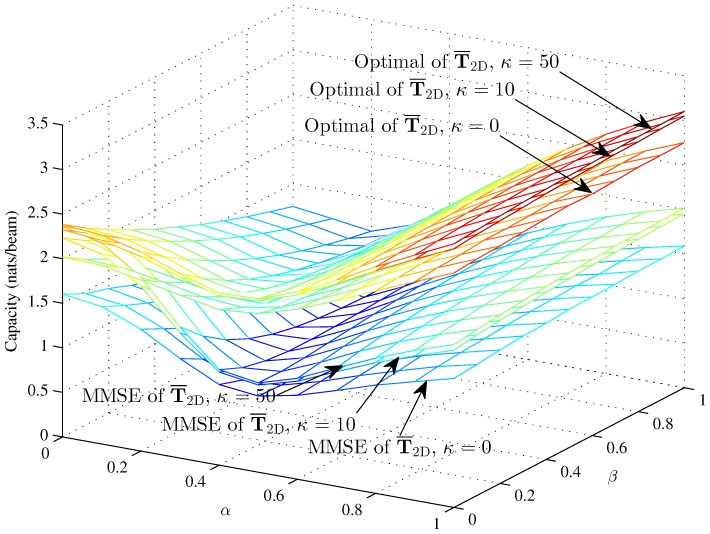
The optimal capacity and MMSE capacity of the hybrid 2D beams with *γ* = 10, *L* = 50 and *M* = 50 (*κ* = 0, *κ* = 10 and *κ* = 50) versus *α* and *β*.

Figure 4, Figure 5 and Figure 6 respectively compare the optimal capacity with MMSE capacity for the hybrid 1D beams, the hybrid 2D beams and modified hybrid 1D beams; in the higher SNR *γ* = 10, the number of sinks is *K* = 1, versus interference factors *α* and *β*.

Obviously, the linear MMSE capacities are lower than the optimal capacity with the same Rician fading factor. The capacities of three models are approximately 2.38 nats/beam when α=0, β=0, K=1 and *κ* = 50 in the higher SNR. The performance of this paper is fit to [33] when β=0. Figure 4 and Figure 5 show that the interference factor *β* from the TCNs decreases the capacity of ${\bar{T}}_{1D}$ and ${\bar{T}}_{2D}$ and increases the capacity as *α* increases to one. That is to say, the capacity decreases first and then increases with *α*, while the coordinate values of inflection points (α,β) decrease as *α* and *β* increase. The optimal capacity and linear MMSE capacity respectively increase as the Rician fading factor increases.

However, the MMSE capacity with Rician fading factor *κ* = 10 and *κ* = 50 is larger than the optimal capacity with *κ* = 0 when α<0.2 and almost 0.15 nats/beam less than the optimal capacity with *κ* = 0 when *α* = 0. In the lower region of *α*, *β* decreases the capacity clearly; while in the higher region of *α*, *β* decreases the capacity in an unclear manner. The curve trends indicate that the effects of intra-beam interference factor *β* of the TCNs are associated with the inter-beam interference factor *α* on the IWSMSNs’ performance and is, to some degree, subjected to the higher region of *α*. Meanwhile, the optimal capacity and MMSE capacity respectively differ more in the lower region of *α* and *β* and differ less in the higher region from *κ* = 0 to *κ* = 10 and *κ* = 50. We can see that the optimal capacity and MMSE capacity are converging as *κ* increases from 0–50, due to the fact that the Rician fading is converted to the additive Gaussian noise channel as κ→∞ to get the nonfading channel.

Figure 6 shows that the capacity of the modified model ${\hat{\bar{T}}}_{1D}$ is lower than ${\bar{T}}_{1D}$ and ${\bar{T}}_{2D}$ by considering all of the inter-beam interference in the same cases. Thus, the capacity of ${\hat{\bar{T}}}_{1D}$ decreases obviously with *α* and *β*. *β* decreases the capacity of ${\hat{\bar{T}}}_{1D}$ as *α* increases from 0–1, which indicates that the IWSMSNs are interference limited. The capacity decreases to zero when *β* = 0 and almost decreases to zero in the lower region of *β* as α=1. We can see that the linear MMSE capacity differs by about 0.65 nats/beam from κ=0 to κ=10 in the lower region of the *α* and *β* and no more than 0.2 nats/beam from κ=10 to κ=50 in the lower region of the *α* and *β*. The MMSE capacity gap of *κ* decreases from the lower region of *α* and *β* to the higher region, and *β* deceases the capacity as the same *α*. Comparing the optimal capacity with the MMSE capacity as κ=50, we find that the MMSE capacity is very close to the optimal capacity in the lower region of *α* and *β*. The optimal and MMSE capacity gap increases first, and it is especially clear from α=0.3 to α=0.8 and decreases to zero from α=0.8 to α=1.

**Figure 7:**
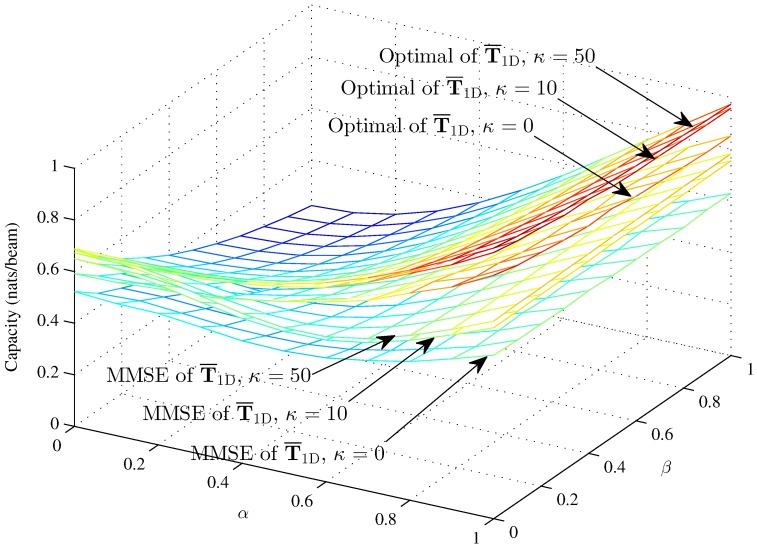
The optimal capacity and MMSE capacity of hybrid 1D beams with *γ* = 1 and *N* = 100 (*κ* = 0, *κ* = 10 and *κ* = 50) versus *α* and *β*.

**Figure 8:**
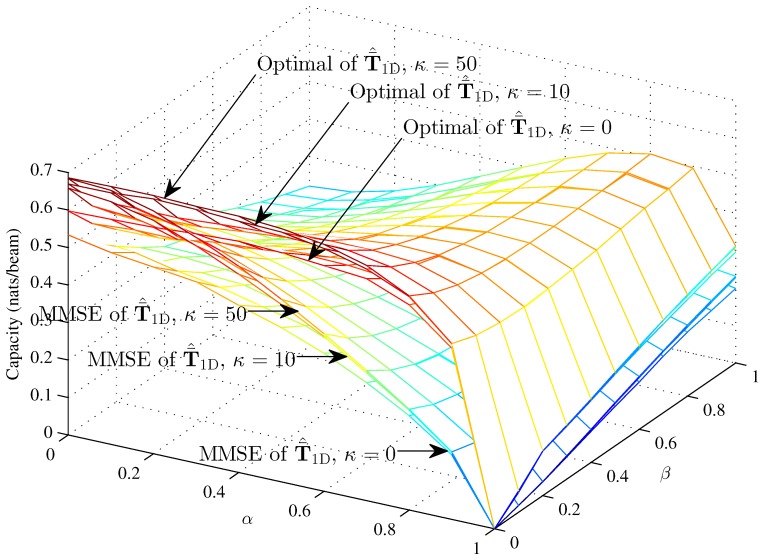
The optimal capacity and MMSE capacity of the modified hybrid 1D beams with *γ* = 1 and *N* = 100 (*κ* = 0, *κ* = 10 and *κ* = 50) versus *α* and *β*.

**Figure 9:**
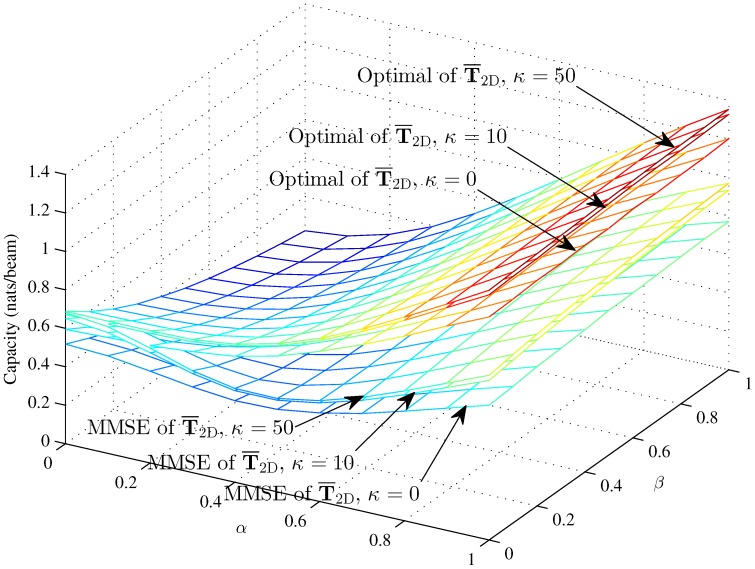
The optimal capacity and MMSE capacity of the hybrid 2D beams with *γ* = 1, *L* = 50 and *M* = 50 (*κ* = 0, *κ* = 10 and *κ* = 50) versus *α* and *β*.

Figure 7, Figure 8 and Figure 9 respectively compare the optimal capacity with MMSE capacity for the hybrid 1D beams, the hybrid 2D beams and modified hybrid 1D beams; in the lower SNR *γ* = 1, the number of sink is *K* = 1, versus interference factors *α* and *β*.

The capacity of three models are approximately 0.69 nats/beam when α=0, β=0, K=1 and κ=50 in the lower SNR. We compare the capacity of the hybrid 1D beams and hybrid 2D beams respectively in the cases of higher SNR and lower SNR between Figure 4 and Figure 7, and Figure 5 and Figure 8. In Figure 7 and Figure 8, *α* progressively increases the optimal capacity and MMSE capacity, while β=0 slightly decreases the capacity with the same *α*, which is different from the curve trends shown in Figure 4 and Figure 5. There, we see that the capacity gap is approximately 0.12 nats/beam from κ=0 to *κ* = 50 when *α* = 1, while the capacity gap is approximately 0.32 nats/beam from κ=0 to *κ* = 50 when *α* = 1. Meanwhile, *β* affects the capacity clearly in the lower region of *α* and in an unclear manner in the higher region of *α*. Figure 9 shows the performance of the modified hybrid 1D beam with γ=1, where the curves approach zero as *α* increases to one. In the lower region of *α*, it is obvious that *β* decreases the capacity; while in the higher region of *α*, *β* affects the capacity in an unclear manner and does not approach zero as *β* increases to one when α=1. In general, the curves of the modified hybrid 1D beam approximately approach zero as *α* and *β* increase.

**Figure 10:**
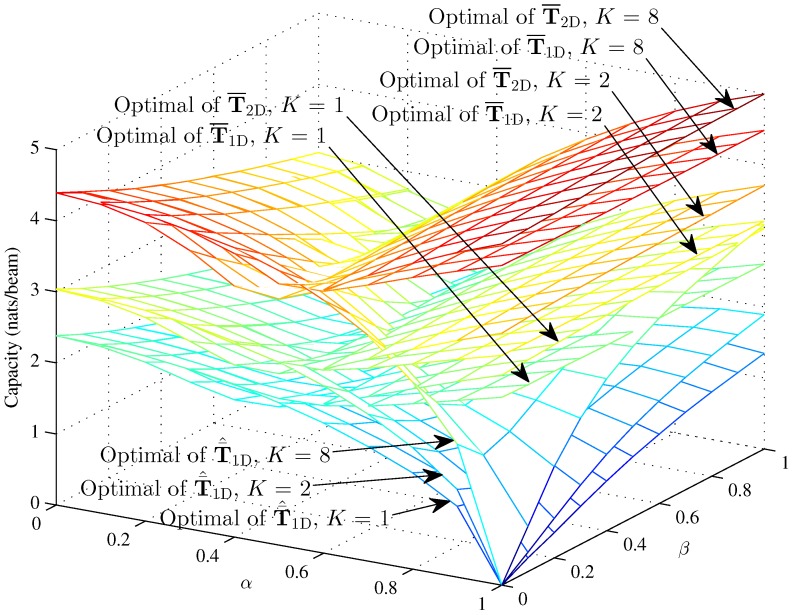
The optimal capacity of the hybrid 1D model and modified hybrid 1D beams with γ=10 and N=100, hybrid 2D model with γ=10, L=50 and M=50 (K=1, K=2, K=8 and κ=50) versus *α* and *β*.

**Figure 11:**
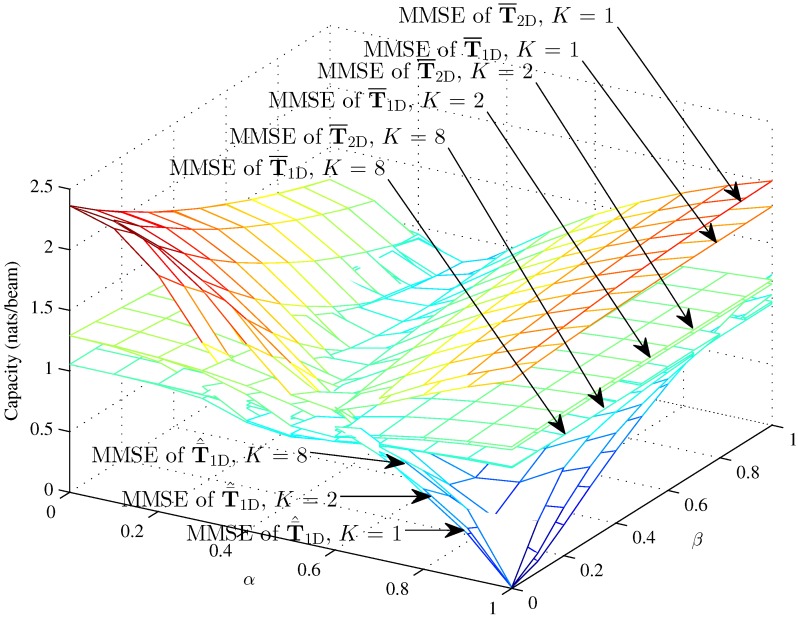
The MMSE capacity of the hybrid 1D model and modified hybrid 1D beams with γ=10 and N=100, hybrid 2D model with γ=10, L=50 and M=50 (K=1, K=2, K=8 and κ=50) versus *α* and *β*.

Figure 10 and Figure 11 respectively show the optimal capacity and MMSE capacity for the hybrid 1D beam, the hybrid 2D beam and modified hybrid 1D beam, in the case of higher SNR γ=10; the number of sinks is K=1, K=2, K=8, and the Rician fading factor κ=50, versus the interference factors *α* and *β*. The optimal capacity of the three models are approximately 3.04 nats/beam and 4.39 nats/beam when K=2 and K=8 with α=0, β=0 and κ=50 in the higher SNR *γ* = 10. However, the linear MMSE capacity of the three models is approximately 1.29 nats/beam and 1.05 nats/beam when K=2 and K=8 with α=0, β=0 and *κ* = 50 in the higher SNR *γ* = 10. Figure 10 indicates that the optimal capacity increases as the number of sinks increases, which rises about 0.66 nats/beam from K=1 to K=2 and about 1.35 nats/beam from K=2 to K=8. However, Figure 11 indicates that the linear MMSE capacity decreases as the number of sinks increases, which reduces about 0.89 nats/beam from K=1 to K=2 and about 0.24 nats/beam from K=2 to K=8 with α=0, β=0 and κ=50 in the lower SNR γ=1. The different tendencies of the optimal and MMSE capacities toward *K* are mainly determined by the decoding strategies. The Rician fading factor is chosen as κ=50, since the capacity in κ=50 can be approximately seen as the upper bound.

Overall, from Figure 10, Figure 11, we can conclude that the MMSE capacity is lower than the optimal capacity and exhibits a different tendency from the optimal capacity towards *K*, which is mainly determined by the single decoding strategy. From Figure 4, Figure 5, Figure 6, Figure 7, Figure 8 and Figure 9, we can see that the intra-beam interference factor *β* affects in an unclear manner the capacity in the higher region of the inter-beam interference factor *α*, which means *α* determines the capacity in the higher region of *α*. The capacity of ${\hat{\bar{T}}}_{1D}$ demonstrates that the IWSMSN is an interference limited network. Meanwhile, the Rayleigh fading causes performance losses for the IWSMSNs, while the strong LOS benefits the performance.

## 5. Conclusions

This paper has proposed the hybrid 1D and 2D beam models, which considered the equivalent interference factor *α* from adjacent beams and the equivalent interference factor *β* from the TCNs intra-beam for the return link of the IWSMSNs. The closed form approximations of the optimal capacity and MMSE capacity were derived under Rician fading. The numerical simulations showed that *β* lowered the capacity, and the capacity of ${\hat{\bar{T}}}_{1D}$ approximately decreased to zero as *α* increased to one and as *β* in the lower region. The effects of intra-beam interference factor *β* of the TCNs were associated with inter-beam interference factor *α* on the performance of the IWSMSNs and was even, to some degree, approaching the higher region of *α*. Due to the LOS and channel correlation characteristic of GEO satellite channels, which were unlike the independent channels of the users from base stations in the TCNs, thus, the capacities of the IWSMSNs were converging as the Rician fading factor *κ* increased, because the Rician fading could be converted to the additive Gaussian noise channel as κ→∞ to get the nonfading channel. The optimal capacity and MMSE capacity exhibited different trends toward the number of sinks *K*, which were mainly determined by using different decoding strategies. Meanwhile, from the numerical simulations, Rayleigh fading caused losses on the performance of the IWSMSNs, while the strong LOS benefited the performance.
